# Supplementary material for: Complexome profiling of the Chlamydomonas psb28 mutant reveals TEF5 as an early PSII assembly factor
Source: Plant Cell. 2025 Mar 17;37(6):koaf055. doi: 10.1093/plcell/koaf055 (PMC12164590; doi:10.1093/plcell/koaf055)
Supplement: koaf055_Supplementary_Data [file koaf055_supplementary_data.zip › psb28_interactive.zip!/plots/intensity/qualityPlots/peptides/CYN38_Cre03.g189800.t1.2.html]

### Cre03.g189800.t1.2

Trivial name: CYN38  
SeqCoverage [median]: 57.60  
Mass: 44.69 kDa  
  
 

| Sequence | MissedCleavage | Mass [Da] | wtIntensity | muIntensity | Score |
| --- | --- | --- | --- | --- | --- |
| ADGFVVQTGDPDGPADGYVDPK | 0 | 2219.00 | 13223.96 | 45246.59 | 114.37 |
| AFGLANIDK | 0 | 947.51 | 0.00 | 1469.27 | 56.01 |
| ASQDVLTR | 0 | 888.47 | 32637.32 | 83390.74 | 184.04 |
| ATLEMK | 0 | 691.36 | 0.00 | 2866.02 | 113.85 |
| AVRASQDVLTR | 1 | 1214.67 | 0.00 | 568.38 | 155.33 |
| DAIIKDFAPEKK | 2 | 1373.76 | 499.06 | 4555.89 | 78.32 |
| DKQAVPLK | 1 | 897.53 | 2262.80 | 9194.39 | 147.34 |
| DKQAVPLKQR | 2 | 1181.69 | 0.00 | 6628.62 | 99.28 |
| DLEGISEALR | 0 | 1101.57 | 11080.33 | 58231.00 | 159.28 |
| DLEGISEALRIPGSK | 1 | 1583.85 | 6464.40 | 26962.28 | 74.77 |
| EALSYVGNIEEAMVK | 0 | 1651.81 | 19863.81 | 69349.40 | 201.66 |
| ESELTPTGSNLLDGR | 0 | 1587.77 | 23579.43 | 90947.14 | 201.00 |
| EYADRPLLLGR | 1 | 1301.71 | 23593.95 | 119437.29 | 96.34 |
| FVDGKENLK | 1 | 1048.56 | 19422.19 | 55204.45 | 132.01 |
| FVDGKENLKNSP | 2 | 1346.68 | 1552.13 | 5665.07 | 76.65 |
| GFPFQVPK | 0 | 918.50 | 690.66 | 6168.38 | 76.85 |
| GFYDGMEVQR | 0 | 1200.52 | 19835.57 | 57574.99 | 197.66 |
| NAAAILR | 0 | 727.43 | 6041.41 | 34169.56 | 114.54 |
| NALPIDNKPIR | 1 | 1249.71 | 32523.46 | 94992.72 | 139.86 |
| NEFENNSASSQVFFLLK | 0 | 1972.95 | 1514.75 | 0.00 | 110.29 |
| QIQKDLEGISEALR | 1 | 1598.86 | 358.74 | 2961.84 | 30.48 |
| QKDAIIKDFAPEKK | 3 | 1629.91 | 1456.10 | 3562.11 | 53.38 |
| RVPFEVR | 1 | 901.51 | 0.00 | 3859.45 | 64.80 |
| SLGPVAR | 0 | 698.41 | 8824.24 | 54257.11 | 130.05 |
| VGDKIEYIK | 1 | 1063.59 | 22619.27 | 66161.26 | 121.73 |
| VPFEVR | 0 | 745.41 | 0.00 | 947.24 | 67.03 |
| VQGDKEPIYDFTLEDLGR | 1 | 2094.03 | 15652.69 | 33129.27 | 138.30 |
|  |  |  | 263696.3 | 937500.4 |  |
